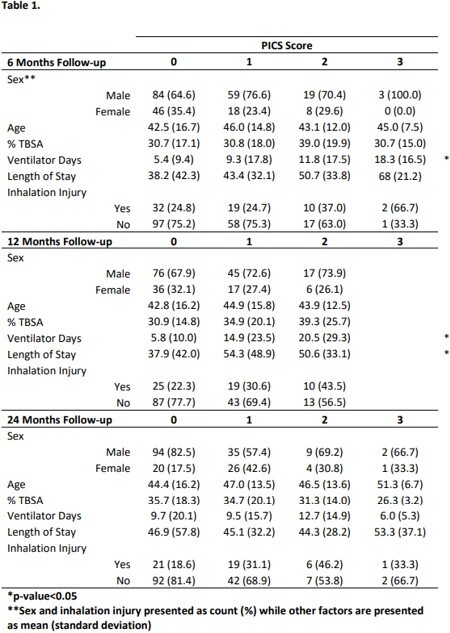# 11 Impact of Post-ICU Syndrome to Return to Work in the Burn Injury Population

**DOI:** 10.1093/jbcr/irae036.011

**Published:** 2024-04-17

**Authors:** Jamie Oh, Alex Morzycki, Caitlin M Orton, Xinyao deGrauw, Sarah A Stoycos, Barclay T Stewart

**Affiliations:** University of Washington, Seattle, WA; UW Medicine Regional Burn Center, Harborview Medical Center, Seattle, WA; Keck School of Medicine, University of Southern California, Los Angeles, CA; University of Washington, Seattle, WA; UW Medicine Regional Burn Center, Harborview Medical Center, Seattle, WA; Keck School of Medicine, University of Southern California, Los Angeles, CA; University of Washington, Seattle, WA; UW Medicine Regional Burn Center, Harborview Medical Center, Seattle, WA; Keck School of Medicine, University of Southern California, Los Angeles, CA; University of Washington, Seattle, WA; UW Medicine Regional Burn Center, Harborview Medical Center, Seattle, WA; Keck School of Medicine, University of Southern California, Los Angeles, CA; University of Washington, Seattle, WA; UW Medicine Regional Burn Center, Harborview Medical Center, Seattle, WA; Keck School of Medicine, University of Southern California, Los Angeles, CA; University of Washington, Seattle, WA; UW Medicine Regional Burn Center, Harborview Medical Center, Seattle, WA; Keck School of Medicine, University of Southern California, Los Angeles, CA

## Abstract

**Introduction:**

Post-ICU Syndrome (PICS) is an important disease entity among survivors of critical injury. PICS consists of three core domains: cognitive, mental, and physical function. Researchers advocate for preventative efforts during a patient’s hospital stay and establishment of post-ICU discharge clinics to screen for and treat PICS-related dysfunction. Despite the recognition of this phenomenon within medical ICU patients, this syndrome has not been characterized within the burn-injured population. We aimed to describe the epidemiology of PICS within the burn population and determine the impact of PICS on patients’ ability to return to work (RTW).

**Methods:**

We reviewed a longitudinal multicenter database for participants (2014 to 2022) with severe burns who completed surveys correlating to the PICS domains at discharge (pre-injury recall), 6, 12, and 24 months after injury. Participants were screened for ICU admission by burn size (≥20% TBSA) or requiring mechanical ventilation. The primary outcome, PICS score, was calculated using PROMIS/VR-12 physical t-scores or ( < 35) for the presence of physical dysfunction, PROMIS/VR-12 mental t-scores ( < 29) for the presence of mental dysfunction, and memory and thought processing difficulty (yes) for the presence of cognitive dysfunction. Each domain was assigned a score of 1 for a total possible score of 3 points . The second outcome, RTW, was defined as those who were previously working or retired and returned to their previous designation or indicated new work post-injury.

Participants demographic and injury characteristics were summarized, and continuous variables were compared with student t-test and reported as mean with standard deviation, and categorical variables were analyzed using χ2 tests and reported as percentages. Mixed-effects Logistic regression modeling was used to examine the impact of PICS on RTW, adjusted for age, sex, burn size, inhalation injury, ventilator days, and length of hospital stay (LOS). Statistical significance was defined as p< 0.05.

**Results:**

Our study included 237 participants. The prevalence of PICS diagnosis remained consistent at each post injury time point (40-45%). There was no statistically significant difference between PICS score and post injury time, age, TBSA, and inhalation injury (Table 1). Mean vent days increased significantly with higher PICS scores at 6 months (p=0.036) and 12 months (p < 0.001) along with mean LOS at 12 months (p=0.048). The odds of RTW significantly decreased by 74% with an increase in PICS score by 1 (adjusted OR: 0.26; 95% CI [0.16-0.42], p< 0.001) (Figure 1).

**Conclusions:**

PICS is common within the severely burn-injured population and serves as an independent predictor of decreased rates of RTW after burn injury.

**Applicability of Research to Practice:**

This work highlights the prevalence of PICS within the burn population demonstrates the need for focused screening and vocational rehabilitation for patients affected by PICS.